# *Echinococcus multilocularis* Infection, Southern Ontario, Canada

**DOI:** 10.3201/eid2502.180299

**Published:** 2019-02

**Authors:** Jonathon D. Kotwa, Mats Isaksson, Claire M. Jardine, G. Douglas Campbell, Olaf Berke, David L. Pearl, Nicola J. Mercer, Eva Osterman-Lind, Andrew S. Peregrine

**Affiliations:** University of Guelph, Guelph, Ontario, Canada (J.D. Kotwa, C.M. Jardine, G.D. Campbell, O. Berke, D.L. Pearl, A.S. Peregrine);; National Veterinary Institute, Uppsala, Sweden (M. Isaksson, E. Osterman-Lind);; Canadian Wildlife Health Cooperative, Guelph (C.M. Jardine, G.D. Campbell);; Wellington-Dufferin-Guelph Public Health, Guelph (N.J. Mercer)

**Keywords:** *Echinococcus multilocularis*, Ontario, southern Ontario, zoonoses, parasites, cestodes, wild canids, foxes, *Vulpes vulpes*, coyotes, *Canis latrans*, dogs, alveolar echinococcosis, public health, prevalence, distribution, Canada

## Abstract

Alveolar echinococcosis, the disease caused by infection with the intermediate stage of the *Echinococcus multilocularis *tapeworm, is typically fatal in humans and dogs when left untreated. Since 2012, alveolar echinococcosis has been diagnosed in 5 dogs, 3 lemurs, and 1 chipmunk in southern Ontario, Canada, a region previously considered free of these tapeworms. Because of human and animal health concerns, we estimated prevalence of infection in wild canids across southern Ontario. During 2015–2017, we collected fecal samples from 460 wild canids (416 coyotes, 44 foxes) during postmortem examination and analyzed them by using a semiautomated magnetic capture probe DNA extraction and real-time PCR method for *E. multilocularis* DNA. Surprisingly, 23% (95% CI 20%–27%) of samples tested positive. By using a spatial scan test, we identified an infection cluster (relative risk 2.26; p = 0.002) in the western-central region of the province. The cluster encompasses areas of dense human population, suggesting zoonotic transmission.

Alveolar echinococcosis (AE) is a chronic infection caused by the larval stage of the *Echinococcus multilocularis* tapeworm and commonly manifests within the liver. In humans and dogs, AE is typically fatal when left untreated. The *E. multilocularis* tapeworm has a wide distribution in the Northern Hemisphere, including extensive endemic regions in North America, Europe, and Asia (*1*), and is usually maintained in a life cycle that involves 2 mammalian hosts. Wild canids (e.g., foxes and coyotes), dogs, and (less commonly) cats act as definitive hosts, which harbor adult parasites in the small intestine without apparent clinical disease. Once mature, adult parasites release eggs, which are shed in the definitive host’s feces. Intermediate hosts (e.g., small rodents) acquire the larval stage by ingestion of infective eggs in the environment. The life cycle is completed when a definitive host consumes an intermediate host containing the larval stage. Humans and dogs can experience AE when eggs of the parasite are consumed. 

In humans, AE is characterized by a lengthy clinical incubation period of 5–15 years, during which the larval stage typically proliferates within the liver, behaving similarly to infiltrative hepatic neoplasia (*2*). Humans with clinical AE cases typically experience cholestatic jaundice, abdominal pain, fatigue, and weight loss (*3*). The preferred treatment is complete excision of parasitic tissue and radical resection of host tissue, depending on the site and size of the lesion, presence of metastases, and patient comorbidities (*4*). Benzimidazole chemotherapy is initiated at the time of diagnosis (*5*). In cases of total surgical resection, treatment is continued for a minimum of 2 years to reduce the likelihood of relapse (*5*). In case-patients who are not surgical candidates, chemotherapy treatment might be prescribed indefinitely to slow the progression of disease (*6*). Historically, in patients from Alaska, France, and Germany, the average survival rate 10 years after diagnosis was 29% when left untreated (*7*). The advent of benzimidazole chemotherapy has increased the 10-year survival rate to ≈80% (*8*).

Red foxes (*Vulpes vulpes*) are commonly the primary definitive host for *E. multilocularis* tapeworms in Europe and North America (*1*). More recently, studies have shown that coyotes (*Canis latrans*) also maintain the parasite in North America (*9*,*10*). This development is important because coyotes can expedite the spread of *E. multilocularis* tapeworms because they have larger home ranges compared with red foxes (*11*).

The area of endemicity of the *E. multilocularis* tapeworm in North America was thought to include 2 distinct regions: the north tundra zone and the north central region. The north tundra zone begins on the west coast of Alaska and extends north and east to occupy most of the Canadian Arctic; the distribution is consistent with that of the Arctic fox (*10*,*12*). The north central region includes the southern portions of the Canada provinces of Alberta, Saskatchewan, and Manitoba, along with 13 neighboring US states (North Dakota, South Dakota, Iowa, Minnesota, Montana, Wyoming, Nebraska, Illinois, Wisconsin, Indiana, Ohio, Missouri, and Michigan) (*9*,*10*,*13*). Recent reports suggest that the distribution is expanding or perhaps is wider than previously thought; for example, in 2009, a dog from the Quesnel region in British Columbia, with no travel history outside of that province, was diagnosed with AE (*14*). A subsequent study determined that ≈33% of wild canids in that region were infected with *E. multilocularis* tapeworms, suggesting a new endemic area (*15*).

Before 2012, Ontario was considered free of *E. multilocularis* tapeworms. Since then, AE has been diagnosed in 5 dogs, 3 privately owned lemurs (*Lemur catta*), and a wild-caught eastern chipmunk (*Tamias striatus*) in the region surrounding the western shores of Lake Ontario in southern Ontario (*16*–*21*; A.S. Peregrine, unpub. data). The primary organ of involvement was the liver in all except 1 case, which involved only a subcutaneous lesion. To the authors’ knowledge, only 1 of the aforementioned dogs had traveled outside this region; the other animals must have acquired the infection locally, probably as a result of ingestion of canid feces containing *E. multilocularis* eggs. Canine AE is a rare disease that most likely occurs when dogs ingest a substantial number of eggs (*22*). Collectively, these cases suggest that parts of southern Ontario have substantial levels of infection among wild canids. 

Although southern Ontario encompasses an extensive geographic area (136,907 km^2^), it is the most densely populated region of the province, with ≈12 million residents (*23*). At the time of the aforementioned cases of AE in animals, human AE was not a disease of public health importance (i.e., it was not reportable) in Ontario; therefore, whether autochthonous human cases were occurring in the province was unknown. Nevertheless, the presence of *E. multilocularis* tapeworms represented a potentially serious threat to human and animal health.

In light of these developments, we sensed an urgent need to accurately define areas in southern Ontario where the *E. multilocularis* tapeworm occurs and to identify the areas of highest risk within this region. We therefore conducted a study to estimate the prevalence and geographic distribution of *E. multilocularis* infection among foxes and coyotes across southern Ontario.

## Materials and Methods

### Carcass Collection and Necropsy

We obtained wild canid carcasses through collaboration with licensed hunters and trappers and the Ontario Ministry of Natural Resources and Forestry. We disseminated information about the project to hunter and trapper groups in southern Ontario that ordinarily harvest coyotes and foxes for their pelts. Submission of a carcass was contingent on provision of the geographic location of origin of the harvested carcass. We obtained carcasses over 2 collection periods: November 1, 2015–August 10, 2016, and August 30, 2016–March 27, 2017. During each collection period, hunters and trappers were limited to 10 carcass submissions of each species. No animals were killed for the purpose of this study. We submitted frozen and fresh carcasses for a limited postmortem examination. We removed the large intestine from each carcass and stored it at −80°C for a minimum of 5 days to eliminate infectivity of the eggs (*24*), after which we collected 2 aliquots of rectal fecal material (3 g each) from each intestinal sample and stored them at −20°C before analysis.

### Magnetic Capture Probe DNA Extraction and Real-Time PCR

At the end of each collection period, we sent fecal samples to the Section for Microbiology at the National Veterinary Institute, Uppsala, Sweden, for analysis using a semiautomated magnetic capture probe DNA extraction and real-time hydrolysis PCR (MC-PCR) method for the presence of *E. multilocularis* DNA (*25*). Compared with the sedimentation and counting technique, which is typically considered the reference standard for the diagnosis of infection in wild canids (*26*), the MC-PCR method is less labor intensive and is well suited for processing large numbers of samples (*25*). When applied to foxes, MC-PCR has an overall sensitivity of 88% (81% with <100 parasites and 96% sensitivity with >100 parasites) and a minimum specificity of 99.9% (*25*,*27*). For each batch of extracted samples that was examined, we used 1 positive and 2 negative controls to validate the extraction process and real-time PCR; the positive control was a known positive fox fecal homogenate. We analyzed all samples in duplicate and considered a sample positive if >1 of the duplicates tested positive.

### Statistical and Spatial Analyses

**Figure 1:**
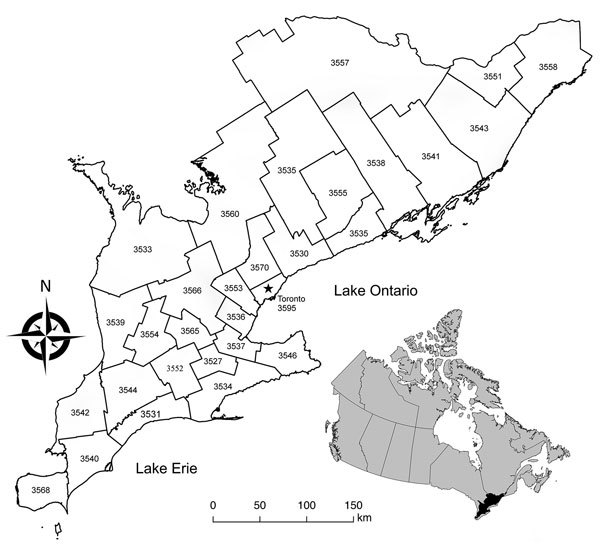
Map of the 29 southern Ontario public health units’ boundaries and corresponding identification numbers (see Table). Inset shows location of southern Ontario within Canada.

Human exposure or case follow-up of AE falls within the legislative mandate for public health in Ontario on the basis of the geographic boundaries of each public health unit (PHU). Therefore, we visualized the prevalence of infection in wild canids across southern Ontario by using choropleth maps organized by the administrative boundaries of the 29 southern Ontario PHUs (Figure 1). To account for potentially unreliable prevalence estimates in certain PHUs resulting from small sample sizes, we used a Bayesian estimation method with local priors to smooth prevalence estimates (*28*,*29*). We performed Bayesian smoothing by using R 3.4.2 with R packages maptools 0.8–39 and spdep 0.6–13 (R Foundation for Statistical Computing, http://cran.r-project.org). Graphic displays were produced by using QGIS 2.14.3 (http://www.qgis.org).

An underlying assumption for constructing CIs is independence of observations. Our data fail to meet this assumption because we cannot assume that the infection status of a wild canid is independent of others in the population. Thus, the dependence of the observations must be considered. Therefore, we constructed Agresti-Coull CIs for prevalence estimates by using Stata/SE 15.1 (StataCorp, http://www.stata.com) (*30*); this method has been recommended for data that violate the assumption of independence (*31*).

To assess for areas of high risk for infection (hotspots), we performed a 1-tailed spatial scan statistic by using a Bernoulli probability model with SaTScan 9.4.4 (https://www.satscan.org). We set the maximum size of the circular scanning window size to 50% of the total population. We estimated the statistical significance of the spatial clusters by using Monte Carlo hypothesis testing based on 999 iterations. We reported statistically significant primary and secondary nonoverlapping spatial clusters. We set the significance level for all analyses at 5% (α = 0.05). We performed the spatial scan test with the observations georeferenced to the centroids of the PHUs and then according to the latitude and longitude of PCR-positive and -negative tested wild canids to assess the consistency of results using different levels of spatial resolution.

## Results

**Figure 2:**
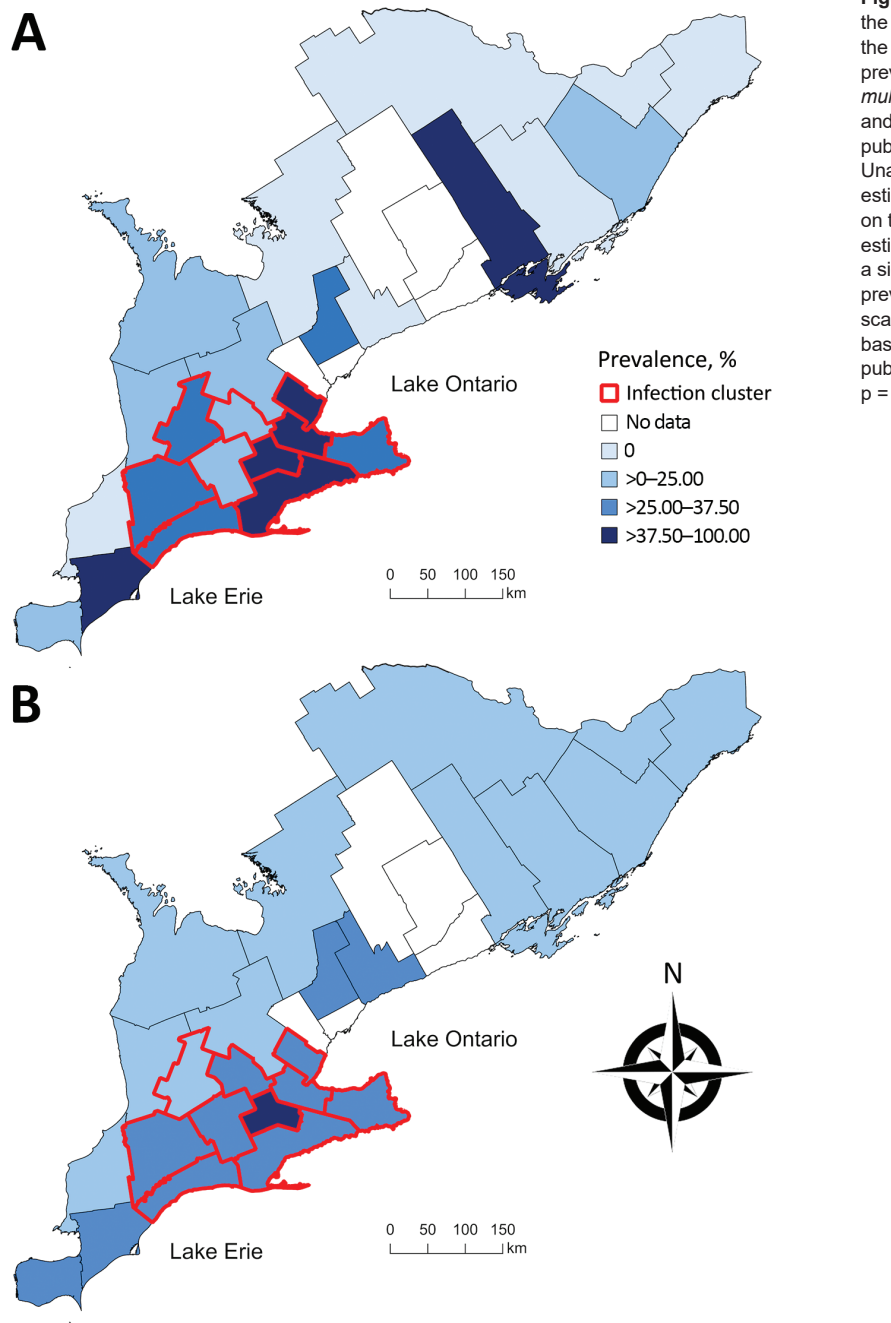
Choropleth maps of A) the unadjusted prevalence and B) the empirical Bayesian-smoothed prevalence of *Echinococcus multilocularis* tapeworms in coyotes and foxes across 25 southern Ontario public health units, 2015–2017. Unadjusted and smoothed prevalence estimates are categorized by quartiles on the basis of unadjusted prevalence estimates. Red boundaries indicate a significant spatial cluster of high prevalence identified by using a spatial scan test with a Bernoulli model on the basis of data georeferenced to their public health units (relative risk 2.26; p = 0.002).

During November 2015–March 2017, we collected 460 wild canids (416 coyotes and 44 foxes) from 25 of the 29 southern Ontario PHUs and tested them for the presence of *E. multilocularis* DNA by using MC-PCR. We collected 205 wild canids (183 coyotes and 22 foxes) in the first collection period and 255 wild canids (233 coyotes and 22 foxes) in the second. During both collection periods, we collected >80% of the wild canids during the months of January, February, and March. Hunters and trappers consistently reported low fox population numbers throughout the duration of the project, resulting in a low number of sampled foxes. No canids were collected from PHUs 3535, 3553, 3555, and 3595 (Figure 1). Overall, 23% (95% CI 20%–27%) of wild canids, from 18 PHUs, tested positive for *E. multilocularis* (Table). Among coyotes, 24% (95% CI 20%–28%) tested positive; 21% (95% CI 11%–35%) of foxes tested positive. Raw prevalence ranged from 0% to 100% among PHUs (Figure 2). Smoothed prevalence by PHU estimates ranged from 4% to 46% (Table) and varied on a gradient of higher to lower prevalence from the southwestern to northeastern regions of southern Ontario (Figure 2).

**Table:** Prevalence of *Echinococcus multilocularis* infection and Bayesian-smoothed prevalence estimates in wild canids, by public health unit, southern Ontario, 2015–2017*

ID	Public health unit	No. wild canids			Prevalence	
		Tested	Positive		Unadjusted, % (95% CI)†	Bayesian estimate, %
3527	Brant County Health Unit	14	10		71 (45–89)	46
3530	Durham Regional Health Unit	2	0		0 (0–71)	27
3531	Elgin-St. Thomas Health Unit	15	5		33 (15–58)	30
3533	Grey Bruce Health Unit	56	6		11 (5–22)	13
3534	Haldimand-Norfolk Health Unit	10	4		40 (17–69)	36
3535	Haliburton, Kawartha, Pine Ridge District Health Unit	0	NA		NA	NA
3536	Halton Regional Health Unit	11	5		45 (21–72)	28
3537	City of Hamilton Health Unit	12	5		42 (19–68)	34
3538	Hastings and Prince Edward Counties Health Unit	1	1		100 (17–100)	25
3539	Huron Health Unit	39	3		8 (2–21)	19
3540	Chatham-Kent Health Unit	1	1		100 (17–100)	31
3541	Kingston, Frontenac and Lennox and Addington Health Unit	2	0		0 (0–71)	5
3542	Lambton Health Unit	1	0		0 (0–83)	21
3543	Leeds, Grenville and Lanark District Health Unit	44	2		5 (<1–16)	4
3544	Middlesex-London Health Unit	41	14		34 (21–50)	28
3546	Niagara Regional Health Unit	19	6		32 (15–54)	37
3551	City of Ottawa Health Unit	3	0		0 (0–62)	4
3552	Oxford County Health Unit	36	7		19 (9–35)	28
3553	Peel Regional Health Unit	0	NA		NA	NA
3554	Perth District Health Unit	35	10		29 (16–45)	24
3555	Peterborough County-City Health Unit	0	NA		NA	NA
3557	Renfrew County and District Health Unit	1	0		0 (0–83)	5
3558	Eastern Ontario Health Unit	1	0		0 (0–83)	4
3560	Simcoe Muskoka District Health Unit	1	0		0 (0–83)	17
3565	Waterloo Health Unit	12	3		25 (8–54)	28
3566	Wellington-Duferin-Guelph Health Unit	55	12		22 (13–35)	20
3568	Windsor-Essex County Health Unit	40	10		25 (14–40)	27
3570	York Regional Health Unit	8	3		38 (13–70)	27
3595	City of Toronto Health Unit	0	NA		NA	NA

*NA, not applicable. †Our data failed to meet the underlying assumption of independence for constructing CIs. We constructed Agresti-Coull confidence intervals for prevalence estimates because this method has been recommended for data that violate the assumption of independence (*31*).

The spatial scan test, georeferenced by PHU, detected a significant spatial cluster of high prevalence of infection (relative risk 2.26; p = 0.002) centered in PHU 3534, consisting of 10 contiguous PHUs (3527, 3531, 3534, 3536, 3537, 3544, 3546, 3552, 3554, and 3565) (Figure 2). The prevalence of infection among the 205 wild canids included in the cluster was 34% (95% CI 28%–40%). A second spatial scan test, using data georeferenced to each wild canid’s location of origin, detected a significant spatial cluster of high prevalence of infection (relative risk 2.53; p = 0.001), with a radius of 120 km also centered in PHU 3534. The prevalence of infection among the 216 wild canids included in the cluster was 34% (95% CI 28%–41%). No statistically significant nonoverlapping secondary high-risk spatial clusters were identified at either level of spatial resolution.

## Discussion

This report describes the prevalence of *E. multilocularis* infection in wild canids in Ontario. Because Ontario was previously considered free of the *E. multilocularis *tapeworm, we were surprised that 23% of the wild canids (107/460) tested positive for the parasite in our study. This finding is comparable to recent wild canid prevalence data from Edmonton, Alberta, Canada (*9*), where the *E. multilocularis* tapeworm has been recognized for decades (*32*). We anticipated that infection among wild canids would be confined to the region surrounding the western shores of Lake Ontario in southern Ontario, where the aforementioned cases of AE were observed. However, our findings indicate that *E. multilocularis* infection in wild canids is widely distributed across the western, central, and eastern regions of southern Ontario, with a high prevalence hotspot consisting of 10 PHUs in the western-central region (Figure 2). The combination of the high prevalence and wide geographic distribution of infection suggests that the *E. multilocularis* tapeworm was not a recent introduction into Ontario. In addition, to the authors’ knowledge, only 1 other study has investigated *E. multilocularis* tapeworms in the province; a survey of 302 red foxes from southern Ontario during 1979–1980 did not detect evidence of the parasite (*33*). Therefore, *E. multilocularis* tapeworms probably were introduced sometime after 1980.

How *E. multilocularis* tapeworms were introduced into Ontario is unclear. However, the spatial pattern of high infection prevalence among wild canids in the southern PHUs that border the northern shores of Lake Erie (Figure 2) might indicate a natural northeastern expansion from Michigan, a known endemic area (*33*). Also, the importation of dogs from endemic areas in North America or Europe, without any requirement for cestocide treatment, might have contributed to the introduction of *E. multilocularis* tapeworms into the province. Notably, molecular characterization of the metacestode stage from 1 of the southern Ontario dogs diagnosed with AE without travel history was consistent with *E. multilocularis* tapeworms of possible European origin (*1*), whereas another appeared to be North American in origin (K. Gesy, pers. comm., 2017 Dec 14). These findings strengthen the possibility that an importation event occurred, perhaps in addition to a natural range expansion. However, the meaning of this information remains unclear because data concerning the epidemiologic importance of individual strain variants are limited (*34*,*35*).

We measured an infection prevalence of 34% (95% CI 28%–40%) among wild canids within the southern Ontario hotspot. Consequently, a question of public health importance is to what extent the human population in southern Ontario is at risk for human AE. Across the endemic countries in Europe, where the prevalence of *E. multilocularis* infection in wild canids ranges from <1% to >50% (*1*), human AE is rare; the overall average annual incidence in these countries ranges from 0.03 to 0.3 cases/100,000 residents (*36*). However, substantial variation in risk exists across regions. For example, in areas with consistently high prevalence in wild canids (i.e., 35%–65% prevalence), the annual incidence of human AE can be as high as 8.1 cases/100,000 residents (*37*,*38*), which is similar to the prevalence estimates among wild canids in the southern Ontario hotspot that we describe. Furthermore, the location of the infection cluster encompasses multiple urban areas with human population densities of up to 1,700 residents/km^2^ (*23*). Therefore, transmission of *E. multilocularis* tapeworms should be considered a public health risk.

In areas endemic for *E. multilocularis* tapeworms, dog ownership has been associated with increased risk for human AE (*37*,*39*–*41*). Dog ownership might entail various human and dog behaviors that might lead to an increased risk for human infection with *E. multilocularis* tapeworms. These behaviors include leaving dogs outside unattended, walking dogs without a leash, allowing dogs to consume rodents, and inconsistent deworming of dogs (*40*). As such, monthly treatment with praziquantel is recommended for dogs that consume rodents in AE-endemic areas to prevent patent intestinal infections and therefore mitigate the risk for transmission to humans (*36*). The same is also recommended for dogs with hepatic AE because such dogs might also have concurrent intestinal infections (*42*). Thus, even in instances of canine hepatic AE, a follow-up investigation of possible exposure to *E. multilocularis* tapeworms for in-contact humans is warranted (*43*).

As of January 1, 2018, *E. multilocularis* infection was designated a reportable disease in animals in Ontario (*44*). Veterinarians and diagnostic laboratories are required to report animal cases directly to their local PHUs to minimize potential risks to human and public health. Furthermore, as of May 1, 2018, *E. multilocularis* infection in humans was designated a disease of public health importance (i.e., a disease that must be reported) in Ontario (*45*). Although human AE was not reportable before 2018, data from the Canadian Institute for Health Information indicate that >3 cases of human AE have been diagnosed in Ontario since 2014 (*46*); however, these data do not include information regarding patient travel or exposure histories. Therefore, whether these cases were locally acquired is unknown. Designating *E. multilocularis* infection as reportable in humans and animals is potentially important because, in AE-endemic areas (i.e., Europe), a large proportion of the economic burden associated with human AE is attributable to patients typically being diagnosed in the late stages of the disease, requiring lifelong chemotherapy and occasionally interventional procedures (e.g., percutaneous biliary and centroparasitic abscess drainage) (*5*,*36*). Therefore, the ability to anticipate *E. multilocularis* exposure and to diagnose early-stage human AE is essential to reduce the need for long-term treatment, thereby minimizing the economic burden associated with the disease. A limitation of having the infection reportable only in humans is that, given the long clinical incubation period of AE in humans, other persons potentially at risk would likely have been infected years earlier. Thus, in areas where *E. multilocularis* infection is endemic, a One Health surveillance approach that also requires mandatory reporting of *E. multilocularis* infection in animals to public health authorities could improve rates of prompt investigation of suspected exposure in persons and lead to earlier diagnosis.

Our study has several limitations. First, sample collection depended on carcass submission from hunters, trappers, and the Ontario Ministry of Natural Resources and Forestry. Although this convenience sampling allowed us to achieve a large sample size, it resulted in underrepresentation of parts of the study area. Because this approach resulted in PHUs with low sample sizes and thus potentially unreliable prevalence estimates, we used a Bayesian estimation method to smooth prevalence estimates. Second, the MC-PCR method used to detect *E. multilocularis* DNA is imperfect, having an overall sensitivity of 88% and specificity of 99% (*25*,*27*). However, we chose to employ this method because it is suitable for large-scale screening and its performance is comparable to what is considered the reference standard for diagnosis of infection in wild canids, the sedimentation and counting technique (*26*).

Our findings underscore the importance for continued surveillance among wild canids within and outside endemic areas in North America to monitor the spread of *E. multilocularis* tapeworms. In addition, an understanding of the prevalence of intestinal infections among dogs in AE-endemic areas would provide valuable information on potential exposure risk in human populations. Collectively, the data would guide public health and veterinary efforts in the development of targeted prevention strategies in this region.
